# Technical note: Institutional solution of clinical cine MRI for tumor motion evaluation in radiotherapy

**DOI:** 10.1002/acm2.13650

**Published:** 2022-05-26

**Authors:** Taeho Kim, Yu Wu, Zhen Ji, H. Michael Gach, Nels Knutson, Stacie Mackey, Matthew Schmidt

**Affiliations:** ^1^ Department of Radiation Oncology Washington University School of Medicine St. Louis Missouri; ^2^ Departments of Radiology and Biomedical Engineering Washington University in St. Louis St. Louis Missouri; ^3^ Department of Radiation Oncology Barnes Jewish Hospital St. Louis Missouri

**Keywords:** cine MRI, Eclipse, tumor motion correlation, tumor motion evaluation

## Abstract

**Purpose:**

Since 4D‐MRI is inadequate to capture dynamic respiratory variations, real‐time cinematographic (cine) MRI is actively used in MR‐guided radiotherapy (MRgRT) for tumor motion evaluation, delineation, and tracking. However, most radiotherapy imaging platforms do not support the format of cine MRI from clinical MRI systems. This study developed an institutional solution of clinical cine MRI for tumor motion evaluation in radiotherapy applications.

**Methods:**

Cine MRI manipulation software (called Cine Viewer) was developed within a commercial Treatment Planning System (TPS). It consists of (1) single/orthogonal viewers, (2) display controllers, (3) measurement grids/markers, and (4) manual contouring tools.

**Results:**

The institutional solution of clinical cine MRI incorporated with radiotherapy application was assessed through case presentations (liver cancer). Cine Viewer loaded cine MRIs from 1.5T Philips Ingenia MRI, handling MRI DICOM format. The measurement grids and markers were used to quantify the displacement of anatomical structures in addition to the tumor. The contouring tool was utilized to localize the tumor and surrogates on the designated frame. The stacks of the contours were exhibited to present the ranges of tumor and surrogate motions. For example, the stacks of the tumor contours from case‐1 were used to determine the ranges of tumor motions (∼8.17 mm on the *x*‐direction [AP‐direction] and ∼14 mm on the *y*‐direction [SI‐direction]). In addition, the patterns of the displacement of the contours over frames were analyzed and reported using in‐house software. In the case‐1 review, the tumor was displaced from +146.0 mm on the *x*‐direction and +125.0 mm on the *y*‐direction from the ROI of the abdominal surface.

**Conclusion:**

We demonstrated the institutional solution of clinical cine MRI in radiotherapy. The proposed tools can streamline the utilization of cine MRI for tumor motion evaluation using Eclipse for treatment planning.

## 1 INTRODUCTION

Magnetic resonance imaging (MRI) is a highly desirable imaging modality compared to CT in radiotherapy because of MRI's superior soft‐tissue contrast without ionizing radiation.^1^ 4D‐MRI (respiratory‐correlated or time‐resolved) was introduced to the radiotherapy workflow in the place of 4D‐CT to characterize the motion of mobile tumors and organs in the abdominothoracic regions.^2^, ^3^

To continuously irradiate the mobile tumor, the range of tumor motion, including a cycle‐to‐cycle variation of the breathing motion, must be included in radiotherapy target volumes.^4^ Since 4D‐MRI is not adequate to capture dynamic respiratory variations, real‐time cinematographic (cine) MRI is actively used in MR‐guided radiotherapy (MRgRT) for tumor motion evaluation, delineation, and tracking.^5^, ^6^, ^7^, ^8^, ^9^

Most radiotherapy imaging display platforms, however, do not support the file format of cine MRI from clinical MRI systems. Radiotherapy practice relies on MRI vendors’ display tools, resulting in limited application of cine MRI. For example, currently, Velocity™ cancer imaging software and Eclipse™ Treatment Planning software (Varian Medical Systems Inc., CA, USA) do not natively handle the format of cine MRI from clinical MRI simulators. In addition, the cine MRI of MRIdian system (ViewRay Inc., Ohio, USA) is not currently compatible with other radiotherapy applications.

The study developed an institutional solution of clinical cine MRI for radiotherapy applications. The institutional solution included (1) tumor motion evaluation and (2) tumor contouring on cine MRI in a commercial treatment planning system. In addition, we developed supplementary in‐house software to assess (3) tumor motion correlation with surrogates.

## 2 METHODS

### 2.1 Cine MRI to Eclipse

For MRgRT, clinical cine MRIs were routinely acquired from a 1.5T Ingenia MRI (Philips Healthcare, Amsterdam, the Netherlands) in our institution. The anterior and posterior receiver array coils were in place for abdominothoracic MRI. Regular free‐breathing 2D images in sagittal, coronal, and orthogonal planes (alternating sagittal‐coronal planes) were acquired using the steady‐state free precession MRI pulse sequence (Balanced Fast Field Echo: BFE) with an imaging field of view = 350 × 350 × 7 mm^3^, voxel size = 2.73 × 2.73 × 7 mm^3^, and total acquisition time/frame ≈250 ms. The acquisition rates were 4 FPS (frames per second) for sagittal and coronal orientations and 3.7 FPS for the orthogonal orientation for real‐time imaging. Sequence parameters are reported in Table 1 in detail.

**TABLE 1:** Cine MRI sequence parameters

Parameters	2D Sag cine 4 FPS	2D Cor cine 4 FPS	2D Sag + Cor cine 3.7 FPS
FOV (mm)	350 × 350 × 7	350 × 350 × 7	350 × 350 × 7
#Slices	1	1	1
NSA	1	1	1
Acquisition pixel (mm)	3.1 × 3.1 × 7	3.1 × 3.1 × 7	3 × 3 × 7
Reconstruction pixel (mm)	2.73 × 2.73 × 7	2.73 × 2.73 × 7	2.73 × 2.73 × 7
SENSE factor	1	1	1
Foldover Dir	AP	LR	AP/LR
Sequence type	BTFE	BTFE	BTFE
Flip angle	50°	50°	60°
Partial Fourier	100%	100%	62.5%
rBW (Hz/pixel)	1771.5	1771.5	1077.6
Acquisition time (s)	0.25/frame	0.25/frame	0.135/image 0.225–0.269/frame
TE (ms)	1.1	1.1	1.12–1.15
TR (ms)	2.2	2.2	2.3
Acquisition matrix	112 × 113 × 1	112 × 113 × 1	116 × 69 × 1
Repetition frames	238	238	520

FOV, field of view; NSA, number of signal average; rBW, receiving bandwidth; TE, echo time; TR, repetition time; AP, anterior‐posterior; LR, left‐right; Sag, sagittal; Cor, coronal.

Cine MRI manipulation software (Cine Viewer) was developed in the Eclipse Treatment Planning System (Varian Medical Systems, Palo Alto, CA, USA) using the Eclipse Scripting Application Programming Interface (ESAPI, Ver. 15.6, Varian). ESAPI retrieves MRI meta‐data and image pixel information for Cine Viewer by connecting to the patient data model generated via the connection between the TPS database and proprietarily formatted image repository. The Cine Viewer consists of (1) single/orthogonal viewers, (2) display controllers, (3) measurement grids/markers, and (4) manual contouring tools. Two display modes included (1) single plane displays for sagittal or coronal cine MRI and (2) orthogonal plane displays for alternating cine MRIs. The display controller tools include functions for display speed, contrast, and color map to improve cine display.

### 2.2 Evaluating tumor motion on cine MRI using Cine Viewer

The measurement grids and markers were used to quantify the displacement of anatomical structures. The grid tool was used to estimate the displacement of the structure by adjusting the size of the grids depending on the scale of the displacement.

The measurement markers were used to quantify the displacement of the structure using an internal distance calculated by the pixel resolution of the cine MRI. The measurement markers consist of horizontal and vertical markers (rotational position is not allowed). The horizontal markers were used to determine the displacement of the anatomical structure on the *y*‐direction of the display plane. In the same way, the vertical markers were used to determine the displacement on the *x*‐direction of the display plane.

### 2.3 Contouring tumor on cine MRI using Cine Viewer

To improve the evaluation of the tumor motion, the manual contouring tool was used to spot the tumor on the designated frame. In addition, contours of the tumor and surrogates (e.g., diaphragm and/or abdomen) on multiple frames were drawn and exported for further evaluation and analysis.

### 2.4 Analyzing tumor motion correlation with surrogates

In addition to Cine Viewer in Eclipse, in‐house software was developed using MATLAB (Mathworks, MA, USA) to assess the tumor motion in detail. The contours of the tumor and the surrogates on multiple frames were used to quantify the motion range and evaluate the motion correlation between tumors and surrogates.

### 2.5 Clinical feasibility evaluation

Clinical feasibility test of Cine Viewer was performed using cine MRIs of five liver cancer patients (mean age: 67 years, range: 53–89) under IRB approval (retrospective study). Each patient underwent three cine MRI acquisitions as a part of standard care for MRgRT. The total imaging duration for the three cine series was about 3 min (250 ms × 238 frames for the sagittal plane, 250 ms × 238 frames for the coronal plane, and 135 ms × 520 frames for the orthogonal planes).

## 3 RESULTS

The institutional solution of clinical cine MRI incorporated with a radiotherapy application was assessed through five case presentations (five liver cancer patients).

### 3.1 Cine MRI to Eclipse

**FIGURE 1:**
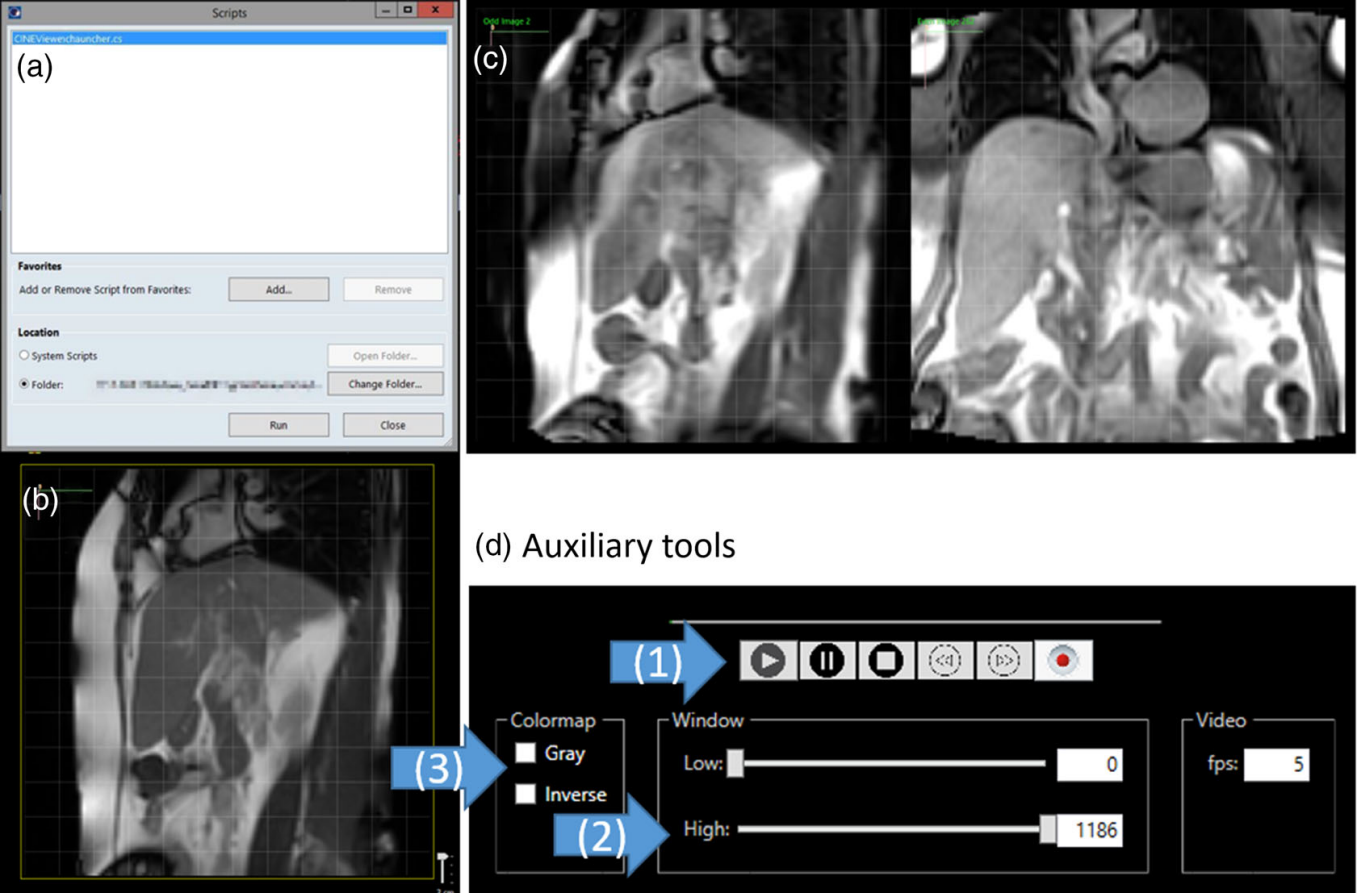
User interface of Cine Viewer in Eclipse. (a) Eclipse script window, (b) single display panel for sagittal cine MRI, and (c) dual display panel for alternating sagittal and coronal cine MRIs. (d) Auxiliary tools include (1) the image playback mode, (2) the image contrast adjustment, and (3) the color map selection

Cine Viewer was launched using the script of Varian Eclipse Scripting Application Programming Interface (ESAPI) in Eclipse shown in Figure 1a. The matrix of the display panel was 600 × 600 for a field of view of 350 × 350 mm^2^. In Figure 1b, sagittal or coronal cine MRIs were loaded on the single display panel with the display controller. The dual display panel displayed alternating cine MRIs in Figure 1c. The display controller includes functions for image contrast adjustment (window/level), color map selection, and image playback mode to improve the cine MRI presentation. The playback mode plays back images to the Cine Viewer that appeared on the MR scanner (image display mode).

### 3.2 Evaluating tumor motion on cine MRI using Cine Viewer

**FIGURE 2:**
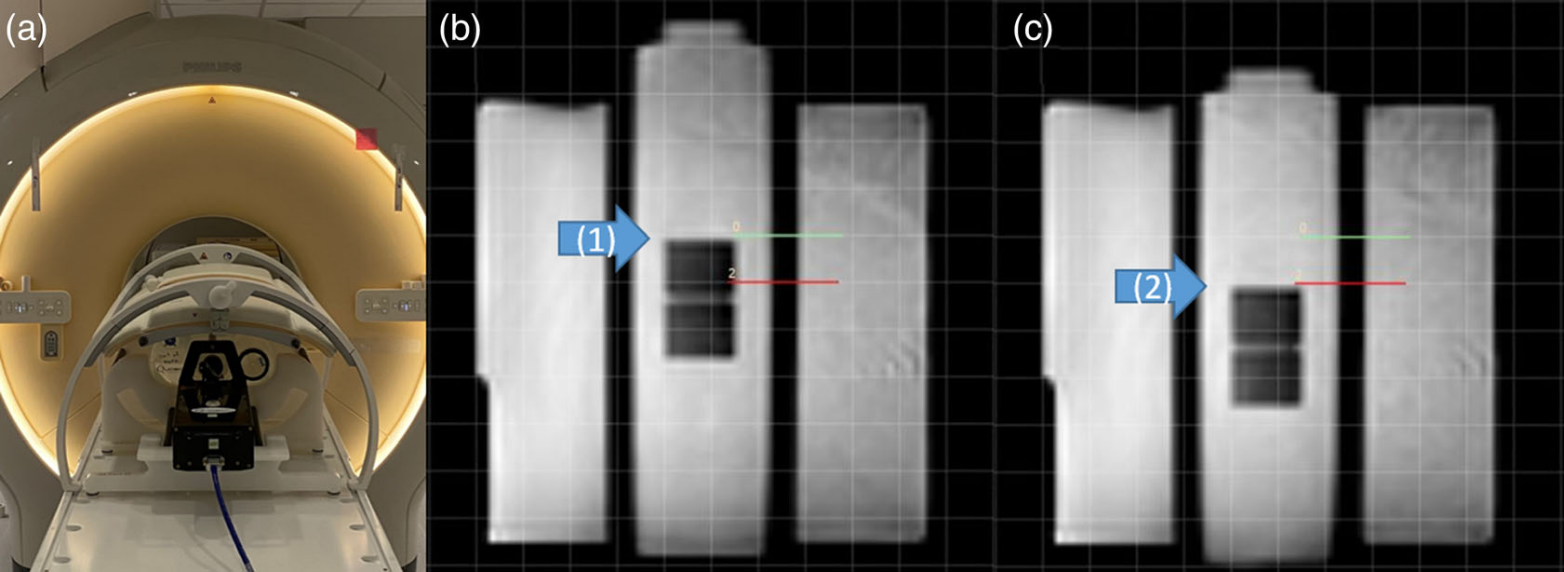
Cine Viewer measurement was verified by measuring the motion phantom with a known motion wave (MRI4D QUASAR motion phantom: 20 mm displacement in 15 cycles per minute of sinusoidal curve). The measurements using the Cine Viewer were 1 grid in 2 cm grids and 19.83 mm from two horizontal markers. (a) Phantom setup, (b) –10 mm excursion, and (c) +10 mm excursion of a cuboid. The arrows indicate the edge of the cuboid

The performance of the Cine Viewer grid and ruler measuring tools was verified. In addition, the software was verified by measuring the motion phantom with a known motion wave (MRI4D QUASAR motion phantom: ModusQA, Ontario, Canada) as shown in Figure 2 (20 mm displacement in 15 cycles per minute of the sinusoidal curve). The Cine Viewer measurements were 1 grid in 2 cm grids and 19.83 mm from two horizontal markers.

**FIGURE 3:**
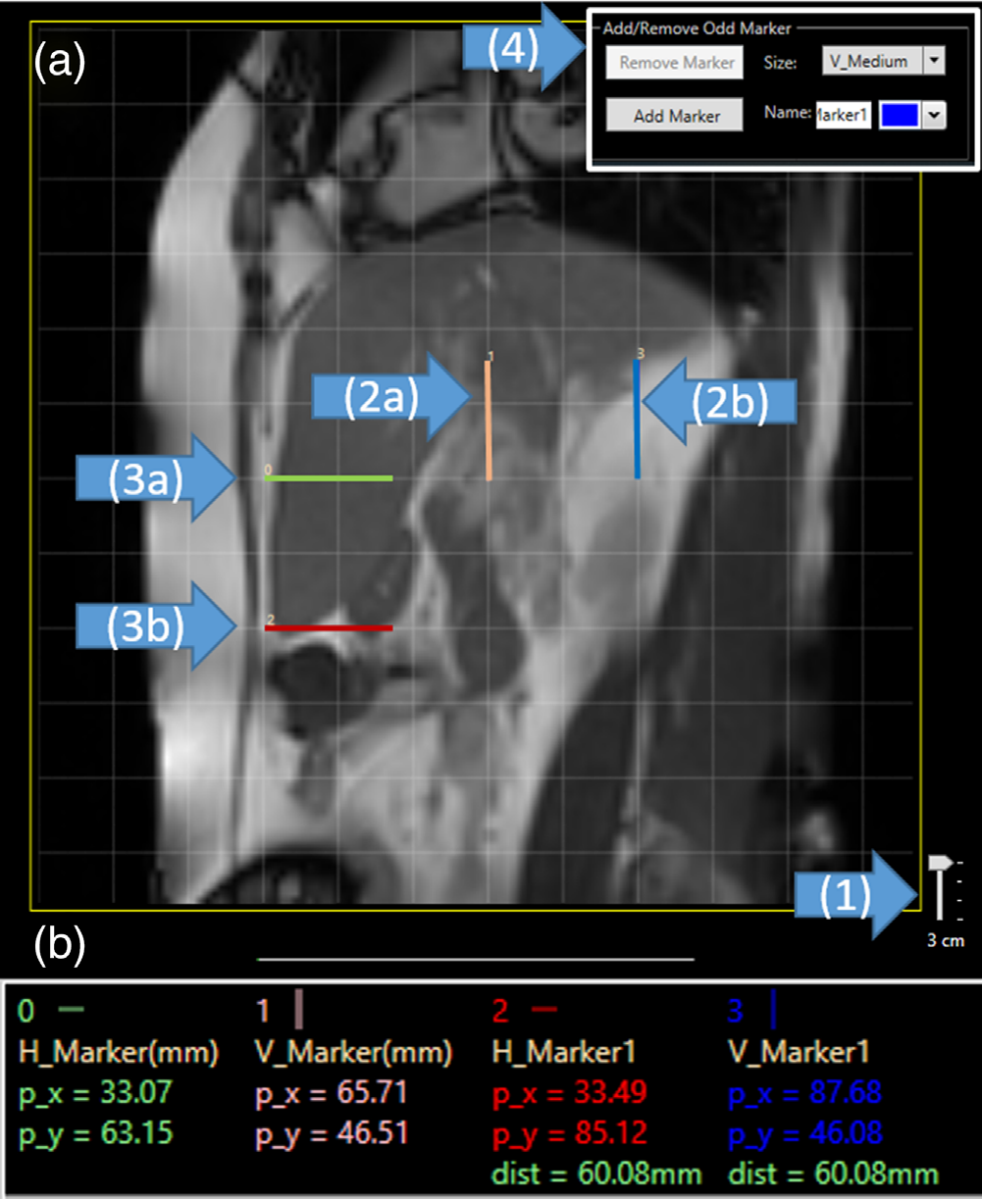
Two measuring tools: grid and marker. (a) Single display panel with 3 cm grid lines and (b) report panels with two horizontal markers (H_Marker) and two vertical markers (V_Marker). p_*x* and p_*y* indicate the pixel location of the markers while dist indicates the distance between the paired markers. (1) Grid scaler (2a and b) vertical markers and (3a and b) horizontal markers and (4) marker controller

In the feasibility study, one of the authors (physicist) contoured based on the clinical target contour drawn by the physician on the 3D MRI in Eclipse. To demonstrate the function of the Cine Viewer such as the stack of the contours, cine frames from about two respiratory cycles (50 frames) were contoured. The tumor motion range can be quantified using two measurement tools: the grid and marker shown in Figure 3. The grid tool was used to qualitatively evaluate the displacement of the structure. The size of the grids can be adjusted from 1 cm to 3 cm (in 1 cm intervals) depending on the scale of the measurements. Since the position of the grid was not adjustable, the grid tool was suitable for the approximation of the motion range.

In contrast, the measurement markers were used to precisely quantify the displacement of the structure as an internal ruler. Initially, one horizontal and one vertical markers were provided. Depending on the measurement direction, an additional marker with a proper name was added using the controller shown in Figure 3a. The other marker was paired with the initial marker, and the distance in the measured direction was reported on the report panel shown in Figure 3b. For example, two horizontal markers were used to determine the distance of the two markers on the *y*‐direction, and two vertical markers were used for the distance of the two markers on the *x*‐direction of the display plane shown in Figure 3a.

Two horizontal markers and two vertical markers were placed on the grid lines. They were separated by two cells of 3 cm‐grid along the *x*‐ and *y*‐direction. The distances on the report panel were 60.08 mm on the *y*‐direction from the horizontal markers and 60.08 mm on the *x*‐direction from the vertical markers, respectively. Since the resolution of the display panel was 0.58 mm (350 mm/600), there was a known limitation in the precision of the measurement.

### 3.3 Contouring tumor on cine MRI using Cine Viewer

**FIGURE 4:**
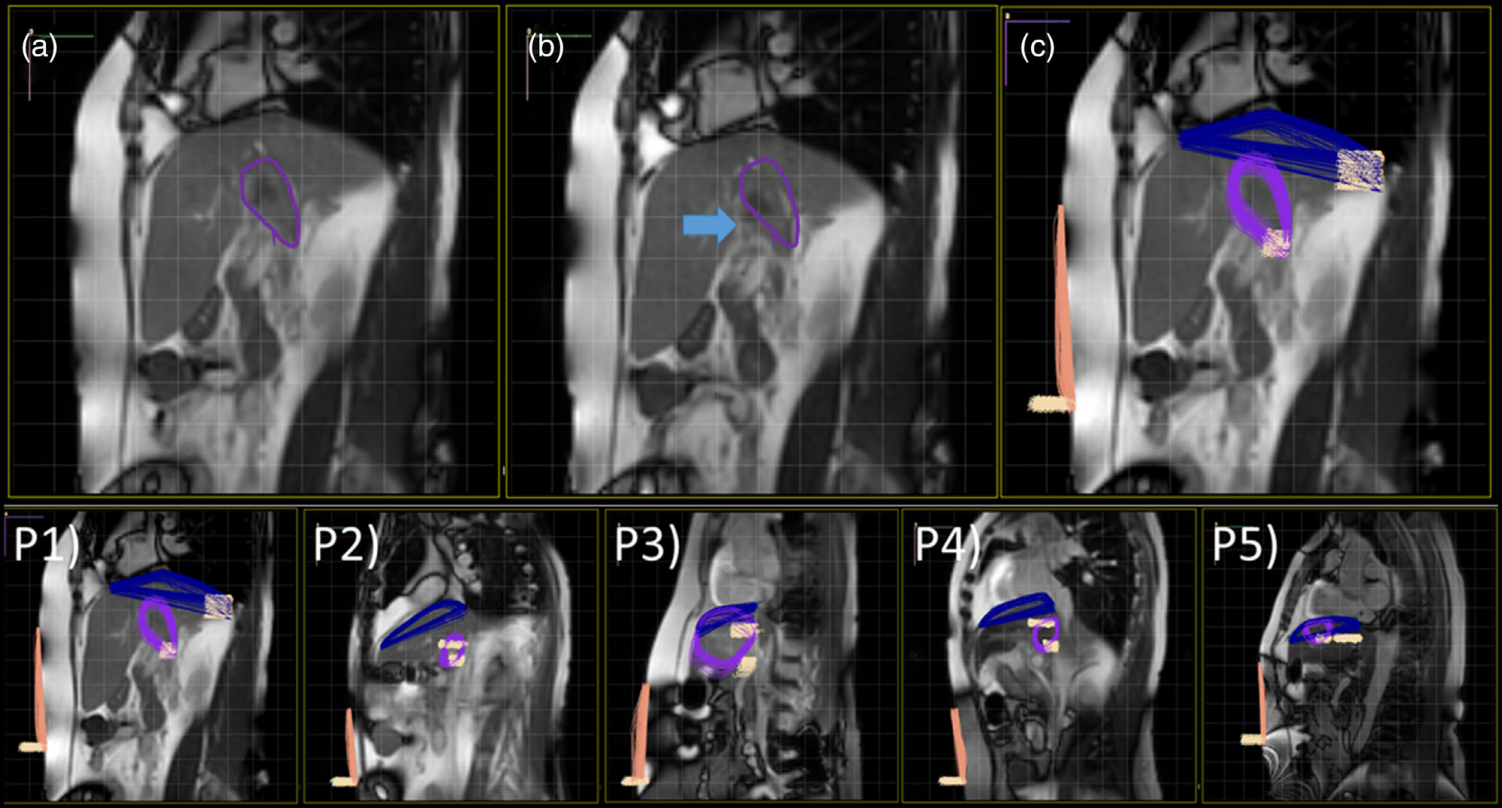
Contours. (a) Tumor contour on the designated frame, (b) through the playback of the cine images, the displacement of the tumor was visualized (blue arrow), and (c) the stacks of the contours were presented to visualize the motion range of the contours (over 50 frames). Purple: tumor, blue: diaphragm, and ivory: abdomen. Lower panel includes five liver cancer patients (p: patient). The ranges of tumor motion using contour excursion in P1 are ∼8.17 mm on the *x*‐direction (AP‐direction) and ∼14 mm on the *y*‐direction (SI‐direction)

The manual contouring tool was utilized to localize the tumor on the designated frame in Figure 4a. Through the playback of the cine images, the displacement of the tumor was visualized and quantified using the measuring tools shown in Figure 4b. In addition to the tumor, the surrogates such as the diaphragm and abdomen on multiple frames were contoured to assess the motion correlation with the tumor. The stacks of the contours are presented in Figure 4c. For example, the stacks of the tumor contours were used to determine the ranges of tumor motion (∼8.17 mm on the *x*‐direction [AP‐direction] and ∼14 mm on the *y*‐direction [SI‐direction] using contour excursion) in Figure 4c. The coordinates of the contours were exported from the Cine Viewer for further motion assessments and analyses. Five liver cancer cases are presented in Figure 4 (P1–P5).

### 3.4 Analyzing tumor motion correlation with surrogates

**FIGURE 5:**
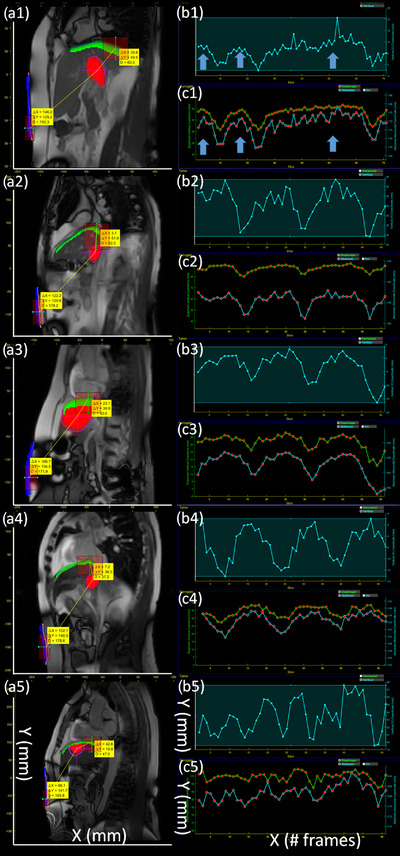
Tumor motion correlation with the surrogates from five cases. (a) Stack of the contours with the distances between the tumor contour and the ROIs of the diaphragm and the abdomen contours. The wave window visualizes patterns of the displacement of (b) the tumor and (c) the surrogate contours (green: diaphragm, blue: abdomen) over frames. To show the motion correlation (arrows), the motion of the tumor in the *y*‐direction along with the diaphragm and the abdomen are exhibited. For example, in (a1), the distance indicates the location of the tumor at +146.0 mm on the *x*‐direction and +125 mm on the *y*‐direction from the ROI (brown boxes) of the abdominal surface. The full ranges of tumor motion using the center of a contour in P1 are ∼6.5 mm on the *x*‐direction (AP‐direction) and ∼17 mm on the *y*‐direction (SI‐direction). The subset number indicates the case number. The *x*‐and *y*‐axes on the image are distances from the image center (mm)

In addition to assessing the tumor motion in the Cine Viewer, the in‐house software was utilized to further visually (qualitatively) examine the motion correlation between the tumor and the surrogate motion as shown in Figure 5a. The contour‐overlap function exhibited the stack of the contours to present the ranges of the surrogate motions in agreement with Figure 4c. The surrogate motions were reviewed to determine a region of interest (ROI).

Once the ROIs of the surrogates were selected, a wave window visualized patterns of the contour displacement over the frame in Figure 5b and c. For example, the displacement of the tumor (the central position of the tumor contour) was presented over the frames (the *x*‐ and *y*‐directions) while the displacement of the diaphragm was on the *y*‐direction and the displacement of the abdomen was on the *x*‐direction. The size of the ROI windows can be adjusted to reduce the signal fluctuation. In addition, the distances between the tumor contour and the ROIs of the surrogates were automatically calculated and presented on the display panel in Figure 5a. This information was used to localize the tumor position based on the surrogate position. For example, the tumor was displaced from +147.0 mm on the *x*‐direction and +118.5 mm on the y‐direction from the ROI of the abdominal surface. Using the graph tool, the tumor motion range utilizing the center of a contour was quantified as ∼6.5 mm on the *x*‐direction (AP‐direction) and ∼17 mm on the *y*‐direction (SI‐direction).

## 4 DISCUSSION

This study presented our institutional solution of clinical cine MRI in Eclipse for tumor motion evaluation. It can be a pertinent approach for utilizing clinical cine MRI in radiotherapy setup. In addition to static 3D volumetric MRI for tumor and/or structure delineation, real‐time cine MRI can benefit motion management and margin determination in treatment planning.^5^

Tumor motion can be evaluated using 4D imaging techniques such as 4D‐CT and 4D‐MRI.^2^, ^3^, ^4^ Respiratory‐correlated 4D imaging techniques suffer from cycle‐to‐cycle variations of the breathing motion, resulting in image artifacts and insufficient motion information.^2^, ^10^ In contrast, time‐resolved 4D‐MRI can be a favorable technique but not clinically applicable yet due to scan time limitations.^2^ Alternatively, 2D cine MRI techniques are actively utilized in radiotherapy since 2D cine MRI can be fast enough to capture dynamic respiratory motion. Therefore, real‐time cine MRI has already been employed in MR‐guided radiotherapy (MRgRT) for tumor delineation and tracking.^5^, ^6^, ^7^, ^8^, ^9^ However, most radiotherapy imaging display platforms do not support the format of cine MRI, and radiotherapy practice relies on MRI vendors’ proprietary tools. For example, cine MRI of ViewRay MRIdian system is only compatible with the vendor's treatment planning system.^8^ Otherwise, custom software disconnected from radiotherapy application has been developed to handle the cine MRI data set.^5^ This approach needs further improvement for the full utilization of clinical cine MRI.

Our institutional solution (Cine Viewer) of clinical cine MRI was developed in the radiotherapy application (Eclipse) using Varian Eclipse Scripting Application Programming Interface (ESAPI). This approach made the tool available to clinical staff, including physicians, physicists, and dosimetrists. Using the solution connects the clinical cine MRI related to the radiotherapy workflow. We demonstrated the primary function of the Cine Viewer, such as motion evaluation using the measuring tools and contouring capability. The dynamic range of the tumor motion can be visualized and determined using the display function shown in Figure 4c, unlike one breathing cycle of respiratory‐correlated 4D imaging. Although the contours on cine MRI cannot be directly transferred to the treatment planning workflow, the range of the dynamic motion can be considered in margin determination.^5^

In addition, we presented the in‐house software to evaluate the motion correlation between the tumor and the surrogates. In beam delivery, the motion correlation between the tumor and surrogate is imperative if used for tumor motion monitoring.^11^, ^12^ Evaluating the motion correlation between the tumor and the surrogates using the ROI in the in‐house software (shown in Figure 5b and c) can help clinical staff such as physicists and radiotherapists to select the proper monitoring ROI during gated beam delivery. The distance between the tumor and the ROI of the surrogate can be used to validate the tumor position in terms of the surrogate position.

The proposed tool has a few limitations. First, the institutional tool only handles 2D tumor motion since 2D cine MRI does not include 3D tumor motion. Although each imaging plane includes the essential motion along the superior‐inferior direction, irregular tumor motion can shift the tumor out of the plane, resulting in different size of the tumor on the imaging plane. In the study, we acquired the orthogonal cine MRIs with 3.7 FPS, which can provide the pseudo‐3D tumor motion information.^13^ Second, as discussed, the 2D contours in the proposed tool cannot be transferred to the 3D treatment planning platform in Eclipse. As Paulson et al. presented, the motion range can be manually applied in margin expansion such as internal margin (IM).^5^ Third, the interfractional variation of the tumor motion cannot be considered using the proposed tool. However, the stack of the tumor motion using the proposed tool can estimate dynamic variations of the tumor motions, while respiratory‐correlated 4D imaging techniques present one cycle of the tumor motion. Fourth, the institutional tool needs further improvement. For example, since the software displays a contour with a contour name, stacking the contours makes a stack of the contour names (Figure 4). In addition, it was not fully automated, such as tumor contouring, motion range measurement, and margin creation from the stacks of the contours. Further improvement and development are ongoing.

The primary function of the proposed tool was discussed through the clinical case presentations. The study demonstrated the clinical feasibility of the proposed tool in a commercial TPS for the tumor motion evaluation in radiotherapy for treatment planning. After further development, tumor motion will be evaluated using the Cine Viewer in our routine clinical workflow. Furthermore, evaluating the correlation between tumors and surrogates can help clinical staff to exploit surface imaging‐based motion management.

## 5 CONCLUSION

We demonstrated the institutional solution of clinical cine MRI in radiotherapy. The proposed tools can streamline the utilization of cine MRI for tumor motion evaluation using a radiotherapy application for treatment planning.

## CONFLICT OF INTEREST

The authors declare no conflicts of interest.

## AUTHOR CONTRIBUTIONS

Kim, Ji, Wu, Knutson, and Schmidt contributed to develop the systems, analysis, and writing of the manuscript. Gach and Mackey contributed to data collection. All authors discussed the results and contributed to the final manuscript.

## Data Availability

The data that support the findings of this study are available from the corresponding author upon reasonable request.
